# Immune responses in rapidly progressive dementia: a comparative study of neuroinflammatory markers in Creutzfeldt-Jakob disease, Alzheimer’s disease and multiple sclerosis

**DOI:** 10.1186/s12974-014-0170-y

**Published:** 2014-10-15

**Authors:** Katharina Stoeck, Matthias Schmitz, Elisabeth Ebert, Christian Schmidt, Inga Zerr

**Affiliations:** Department of Neurology, Clinical Dementia Centre, DZNE and National Reference Centre for Human Prion Diseases, University Medical Center Göttingen, Robert-Koch-Str.40, 37075 Göttingen, Germany

## Abstract

Immunological responses may contribute to disease progression and clinical heterogeneity in neurodegenerative dementia, for example, Alzheimer’s disease (AD) and Creutzfeldt-Jakob disease (CJD). Recently, a rapidly progressive form of AD (rpAD) has been described. On neuropathological grounds classical AD and rpAD are not distinguishable at present. All those protein aggregopathies show a state of chronic inflammation with microglia activation and production of proinflammatory cytokines. In this context, it is hypothesized that the severity of the surrounding inflammation substantially contributes to disease progression and accelerated disease courses as seen in rpAD.

Using a cytokine multiplex array based on Luminex Technology, we studied 17 pro- and anti-inflammatory cytokines in cerebrospinal fluid (CSF) and serum from patients with classical dementia (AD) or rapidly progressive dementia (Creutzfeldt-Jakob disease (CJD), rpAD). For controls, we chose patients with multiple sclerosis (MS) and non-neurodegenerative diseases. We found a significant and isolated elevation of proinflammatory cytokines (IL-13, TNF-α and G-CSF) in the serum of rpAD patients. In CSF, IL-8 and MCP-1 chemokines were significantly elevated in CJD patients and MCP-1 in AD patients.

In conclusion, we found a characteristic proinflammatory cytokine response in the serum of rpAD patients. It might explain the more rapidly progressive course of the rpAD subform and can be helpful in distinguishing between classical AD and rpAD.

**Electronic supplementary material:**

The online version of this article (doi:10.1186/s12974-014-0170-y) contains supplementary material, which is available to authorized users.

## Introduction

Immunological responses in neurodegenerative disease pathogenesis such as in Alzheimer’s disease (AD) and Creutzfeldt-Jakob disease (CJD) have become of increasing scientific interest in the past years. Both diseases are neuropathologically characterized by the deposition of amyloid fibrils formed by amyloid beta (Aβ) in AD, whereas in prion diseases, the depositions are formed by the abnormally folded host-derived prion protein scrapie (PrP^Sc^).

Since the change in the conformation seems to be the major pathogenic event, both diseases are considered as aggregational or conformational disorders.

It has been shown that plaque formation in AD and CJD is influenced early by immune system responses through MHC II surface expression and complement activation, which secondarily leads to microglia recruitment and secretion of proinflammatory cytokines such as interleukin (IL)-1β, IL-6, and tumor necrosis factor-α (TNF-α), as well as chemokines (IL-8, macrophage inflammatory protein-1β (MIP-1β), monocyte chemoattractant protein-1 (MCP-1) and growth factor macrophage-colony stimulating factor (MG-CSF) [1,2].Within the concept of plaque formation, the issue of whether this microglial response is purely reactive, or whether it also has causative effects, arises. Microglial-produced inflammatory mediators have neurotoxic as well as neuroprotective mechanisms. This has been shown, for example, for TNF-α, where excess levels might cause neurotoxicity, and mild oxidative and low-dose TNF-α could, alternatively, trigger neuroprotective or anti-apoptotic pathways [3]. At later stages in the process of neurodegeneration, neuronal loss occurs due to the neurotoxic effects of proinflammatoy immune-mediating molecules [4]. However, it remains unknown whether neurons are active or passive players in the process of neuronal loss, as it has been shown that increased expression of complement factors and inducible cyclooxygenase-2 (COX-2) were mainly found in neurons and only to a minor extent in microglia cells in AD [5].

In addition, an important role of cellular prion protein (PrP^C^) in modulating age-associated brain disorders such as AD has been described. PrP^C^ can either regulate the amyloid beta precursor protein processing (the uptake of Aβ) [6-8], or it may protect cells from inflammation [9]. With PrP^C^ silencing, it has been shown that PrP^C^ plays a role in immunological homoeostasis by regulating the T cell receptor-mediated T cell activation. This opens a novel potential target for therapeutic immunomodulation [10].

Given this background information, we hypothesized that immune response mechanisms such as microglia activation and production of proinflammatory cytokines, as well as altered levels of PrP^C^, might trigger increases in disease severity and rapid courses as seen in rpAD and CJD in the clinical context.

CJD represents a form of a rapidly progressive dementia caused by protein misfolding. The main disease duration generally extends to 6 month [11]. However, different disease courses have been described depending on the molecular subtype, with disease durations ranging from 4 to 24 month [12].

There is also substantial heterogeneity in the clinical presentation in AD patients regarding disease course. In classical AD, mean disease duration of 7 years is acknowledged. However, there is a huge variety in AD, and some patients follow a disease course up to 20 years. In the framework of our surveillance studies, a distinct subtype of AD, rapidly progressive AD (rpAD) was identified. Patients with rpAD can mimic the clinical course of CJD with a progressive cognitive decline (>6 mini-mental test points/year) and short disease duration (<2 years, 6 to 8 months), as well as early focal neurological signs, such as the occurrence of extra-pyramidal symptoms and myoclonus. Patients with rpAD are often similar in age to CJD (age range 60 to 70 years), whereas patients with classical AD are often older at disease onset (age range 70 to 80 years and older) [13].

Although a commonly accepted definition of rpAD does not exist at present, this subtype is subject to present scientific work with regard to the identification of disease-modifying factors.

In the present study we addressed the role of inflammation on the disease course in aggregational neurodegenerative diseases by investigating pro- and anti-inflammatory cytokines in cerebrospinal fluid (CSF) and serum from patients with classical AD, as well as patients with rpAD and CJD, subgroups representative of rapidly progressive dementia. Patients with multiple sclerosis (MS), the most common disease of chronic CNS inflammation that causes neurodegeneration, and non-neurological patients were included as controls.

We focused therefore on pro- and anti-inflammatory cytokine profiles that suggest a specific immune response (for example, TH1, TH2, TH17 or innate immune response). Both serum and CSF samples were analyzed in order to identify either systemic (serum) or CNS-borne (CSF) immune system changes.

## Methods

### Patients and sample collection

We analyzed CSF and serum samples from 12 sporadic CJD patients with various codon 129 genotypes (6 MM, 3 MV and 3 VV; 6 female, 6 male; aged 55 to 85 years; mean age 64.5 ± 1.5 years), collected between 2012 and 2013. We analyzed samples from 35 AD patients (15 rapidly progressive AD, 20 classical AD; 20 female, 15 male; aged between 60 and 85 years; mean age 69.5 ± 2.5 years), collected between 2009 and 2013 and from 12 multiple sclerosis patients (6 female, 6 male; aged 30 to 70 years; mean age 55 ± 4.5 years), collected in 2012. All samples were directly stored at -80°C and repeated freezing/thawing cycles were avoided.

All patients with CJD were classified as definite cases by neuropathological examinations or as probable CJD cases according to diagnostic consensus criteria [11,14,15]. Classical AD patients were diagnosed according to the Dubois criteria [16]. Rapidly progressive AD patients were selected according to clinical presentation as reported previously by the German CJD surveillance group [17]. Risk factors such as homozygosity for ApoE 4, a history of brain trauma, and a current treatment with immunosuppressive drugs, as well as a state of systemic infection at the point of sample collection were excluded. MS patients were diagnosed according to McDonald criteria [18]. Patients of the control group (6 female, 6 male; aged 50 to 85 years; mean age 62.5 ± 2.5 years) were diagnosed with depression, headache, vertigo or pain syndromes. An organic disease of the central nervous system was excluded during the diagnostic workup. All collected CSF and serum samples were stored at -80°C prior to analysis.

### Ethics

The present study was conducted according to the revised Declaration of Helsinki and Good Clinical Practice guidelines and has been approved by the local ethics committee in Göttingen (No. 9/6/0). Informed consent was given by all study participants or their legal next of kin. All samples were anonymized.

### Cytokine multiplex assay

Cytokine levels in CSF and serum samples were measured using the Bio-Plex 200 System, which is based on Luminex xMAP Technology (Bio-Rad, Munich, Germany). In the present study, we screened CSF and serum samples using the Bio-Plex Pro human cytokine 17-plex assay, cytokine group 1 (Bio-Rad, Munich, Germany). The following cytokines were simultaneously detected (detection range is shown in parentheses): IL-1β (3.3 to 3,261 pg/ml), IL-2 (2.1 to 17,772 pg/ml), IL-4 (2.2 to 3,467 pg/ml), IL-5 (3.1 to 7,380 pg/ml), IL-6 (2.3 to 18,880 pg/ml), IL-7 (3.1 to 6,001 pg/ml), IL-8 (1.9 to 26,403 pg/ml), IL-10 (2.2 to 8,840 pg/ml), IL-12 (p70) (3.3 to 13,099 pg/ml), IL-13 (3.7 to 3,137), IL-17 (4.9 to 12,235), G-CSF (2.4 to 11,565), GM-CSF (63.3 to 6,039), IFN-γ (92.6 to 52,719), MCP-1 (2.1 to 2,820) (MCAF), MIP-1β (2.0 to 1,726) and TNF-α (5.8 to 95,484).

The cytokine assay was performed according to the manufacturer’s instructions. Samples were thawed on ice and mixed by vortexing and then diluted 1:4 in sample diluent buffer. The signal was detected by using the Bio-Plex 200 system and the Bio-Plex Manager Software version 6.0.

### Determination of Aβ1-40 and Aβ1-42 level

Levels of Aβ1-40 were ascertained using the full-length Aβ1-40 high sensitive assay ELISA obtained from IBL International (Hamburg, Germany), which had a detection range of 1.56 to 100 pg/ml. Levels of Aβ1-42 were determined by the use of a commercially available ELISA kit (INNOTEST™-AMYLOID (1-42), Innogenetics, Gent, Belgium) with a detection range of 125 to 2,000 pg/ml.

All ELISA measurements were performed according to the protocol of the manufacturer. The colorimetric reaction was measured at 450 nm with a 1420 Victor2 Multilabel Counter (Wallac) (PerkinElmer, Waltham, Massachusetts, USA). Each sample was measured in duplicate. For analysis, we calculated the median.

### Determination of Tau and p-Tau 181 level

CSF levels of total Tau protein were measured using a commercially available ELISA kit (INNOTEST™ hTAU Ag, Innogenetics) with a detection range of 50 to 2,500 pg/ml (detection limit: 34 pg/ml). For the determination of Tau level, we followed the manufacturer’s instructions. Human Tau, phosphorylated at Thr181 (phosphorylated Tau) was analyzed quantitatively by the use of a commercially available ELISA kit (INNOTEST™PHOSPHO-TAU (181 P), Innogenetics) with a detection range of 15.6 to 1,000 pg/ml (detection limit: 3 pg/ml).

Briefly, before antibody incubations, each sample (75 μl) was diluted 1:1 in sample diluent. The colorimetric reaction was measured at 450 nm with a 1420 Victor2 Multilabel Counter (Wallac) (PerkinElmer). Each sample was measured in duplicate. For analysis, we calculated the median.

### Determination of prion protein level

To determine the prion protein (PrP) concentration, we used a commercial BetaPrion BSE-ELISA Test Kit (AJ Roboscreen, Leipzig, Germany) and omitted the PK digestion step. We followed a slightly modified protocol as reported earlier [19,20]. Our aim was to measure the concentration of total PrP in CSF and serum. Samples were diluted and measured according to the manufacturer’s instruction. To confirm the results, a statistically robust number of samples were analyzed by an additional X-MAP-based assay for PrP, which has already been published [19]. All chemicals and equipment were obtained from (Bio-Rad). The detection range was from 6.25 ng/ml to 500 ng/ml.

### Statistical analysis

Statistical evaluation of the data was performed using STATISTICA for Windows version 10 (Stat Soft, Inc.). To determine the statistical significance, we used the nonparametric Kruskal-Wallis test. The statistical analysis was shown for each patient group [see Additional file 1]. Differences were considered significant at an error probability of *P* <0.05.

## Results

### Levels of proinflammatory cytokines were increased in the serum of patients who had a rapidly progressive form of Alzheimer’s disease

**Figure 1:**
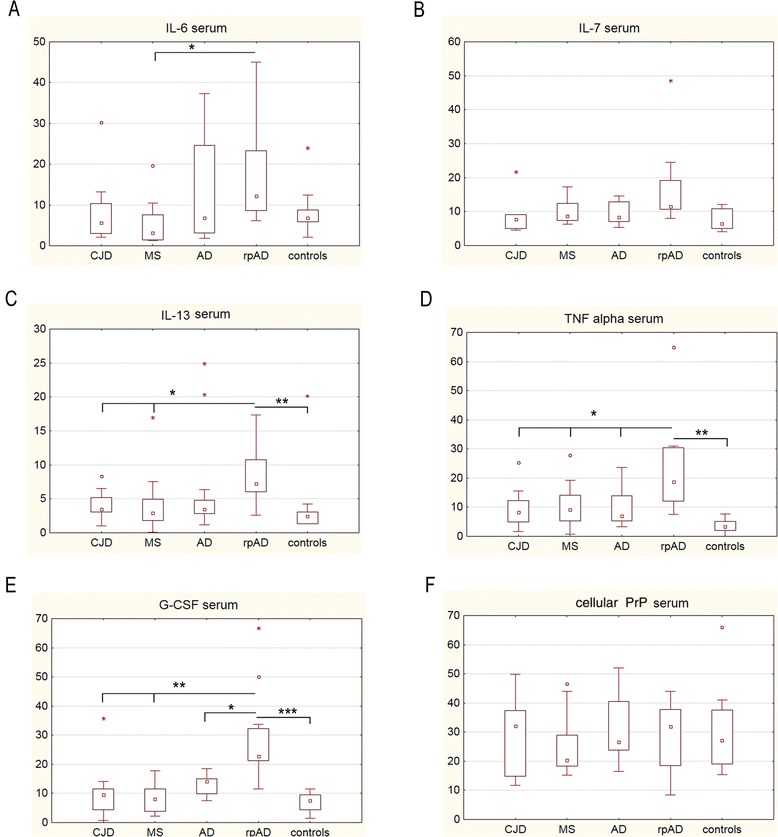
**Different immune responses in the serum of patients with rapidly progressive dementia.** **(A-E)** Profiling of cytokines (measured in pg/ml) in serum from Creutzfeldt-Jakob disease (CJD) (n =12), multiple sclerosis (MS) (n =12), Alzheimer’s disease (AD) (n =20), a rapidly progressive form of AD (rpAD) (n =15) and control (n =12) patients was performed using the Bio-Plex Pro human cytokine 17-plex assay. A significant distinction in rpAD patients was identified for IL-6, IL-13, TNF-α and G-CSF. **(F)** PrP^C^ level in serum did not vary among groups. Columns represent means with SD. Statistics were performed by using the non-parametric Kruskal-Wallis test (+Tukey’s *post hoc* tests). The number of stars indicates the significance level: one star (*) for *P* <0.05, two (**) for *P* <0.01 and three (***) for *P* <0.001.

The levels of a cytokine panel, consisting of 17 different cytokines, were measured in serum samples from CJD, MS, AD, rpAD and control patients using the Bio-Plex Pro human cytokine 17-plex assay. As a major finding we observed significantly elevated concentrations of proinflammatory cytokines (IL-13, TNF-α and G-CSF) in serum of patients with rpAD compared to AD, CJD, MS and control patients. Furthermore, the level of IL-6 was significantly increased in rpAD cases compared to MS patients, while determination of IL-7 level showed a slight increase (not significant) in rpAD patients (Figure 1A-E). The immune response profile was reflected both by TH1/TH17 (IL-6, IL7 and TNF-α) and TH2-associated cytokines (IL-13 and G-CSF).

**Figure 2:**
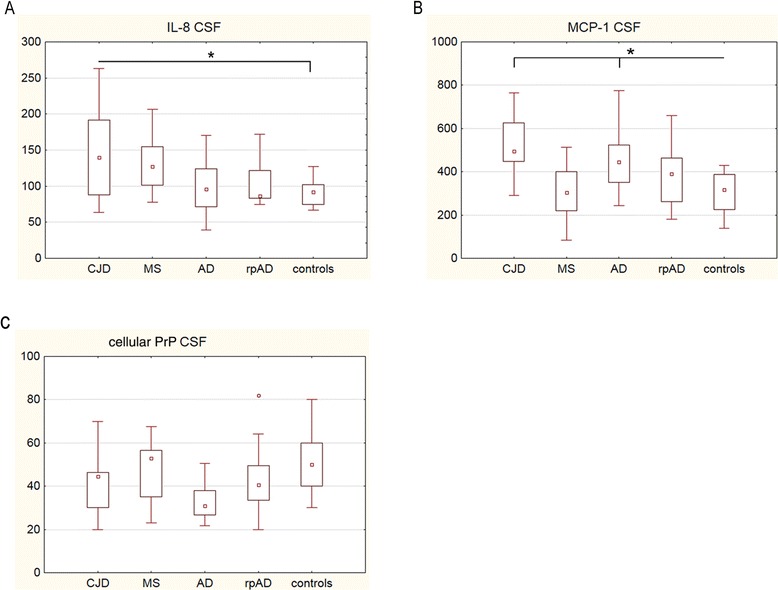
**Different immune responses in cerebrospinal fluid (CSF) of patients with rapidly progressive dementia.** **(A-B)** Profiling of cytokines (pg/ml) in CSF from Creutzfeldt-Jakob disease (CJD) (n =12), multiple sclerosis (MS) (n =12), Alzheimer’s disease (AD) (n =20), a rapidly progressive form of AD (rpAD) (n =15) and control (n =12) patients was performed by using the Bio-Plex Pro human cytokine 17-plex assay. A significant increase of IL-8 was found in CJD patients, while the level of MCP-1 was significantly elevated in CJD and AD patients but not in rpAD patients compared to control donors. **(C)** PrP^C^ level in the CSF did not vary significantly among groups (n =12). Columns represent means with SD. Statistics were performed by using the non-parametric Kruskal-Wallis test (+ Turkey's post hoc tests). The number of stars indicates the significance level: one star (*) for *P* <0.05, two (**) for *P* <0.01 and three (***) for *P* <0.001.

The expression of IL-1β, IL-2, IL-4, IL-5, IL-8, IL-10, IL-12 (p70), IL-17, GM-CSF, IFN-γ, MCP-1 and MIP-1β was either not measurable or not significantly changed in serum of our patient cohort (data not shown). Additionally, we observed no significant variation in levels of PrP^C^ between rpAD, AD, MS, CJD patients and controls in either the serum (Figure 1F) or the CSF (Figure 2C).

### Elevated chemokine levels in cerebrospinal fluid in patients with Alzheimer’s disease and Creutzfeldt-Jakob disease

The levels of a cytokine panel, consisting of 17 different cytokines, were measured in CSF samples from CJD, MS, AD, rpAD and control patients using the Bio-Plex Pro human cytokine 17-plex assay. In CSF, we observed significantly elevated levels of IL-8 und MCP in CJD and AD (MCP-1) patients compared to control donors (Figure 2A and B). Both cytokines are potent proinflammatory chemokines related to an innate immune response. The expression of IL-1β, IL-2, IL-4, IL-5, IL-6, IL-7, IL-10, IL-12 (p70), IL-13, IL-17, G-CSF, GM-CSF, IFN-γ, MIP-1β and TNF-α was either not measurable or not significantly changed in the CSF of our patient cohort (data not shown).

### Comparison of dementia marker profiles in the cerebrospinal fluid of patients with Alzheimer’s disease or a rapidly progressive form of Alzheimer’s disease

**Figure 3:**
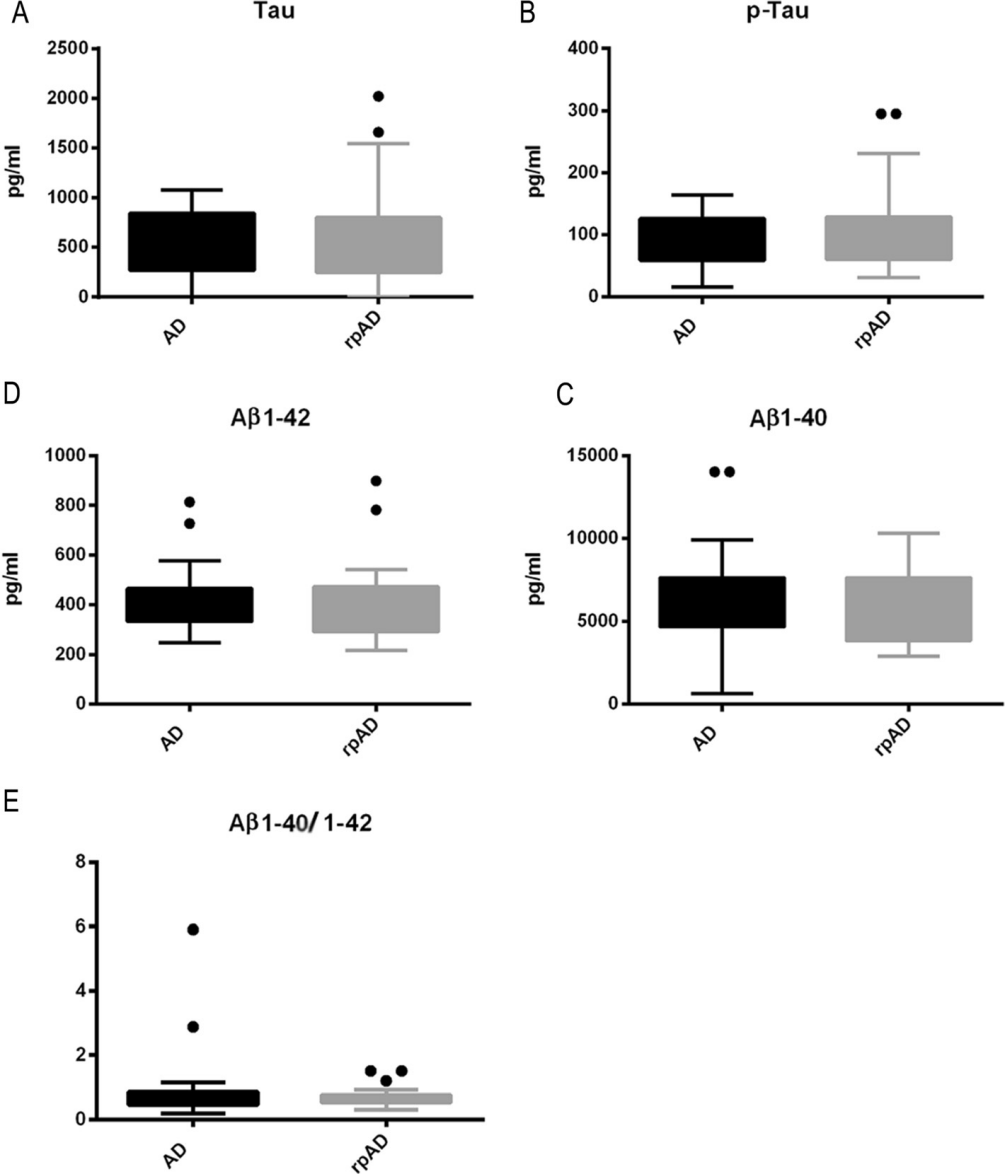
**No differences in the levels of cerebrospinal fluid (CSF) dementia markers between patients with Alzheimer’s disease (AD) and patients with a rapidly progressive form of Alzheimer’s disease (rpAD).** **(A-D)** Expression levels of total Tau, p-Tau, Aβ1-40 and Aβ1-42 in the CSF of AD and rpAD (each, n =8) were measured by ELISA. **(E)** Aβ ratios were calculated from Aβ1-40 and Aβ1-42. No significant variations could be detected in either cohort. Error bars represent means with SD. The number of stars indicates the significance level: one star (*) for *P* <0.05, two (**) for *P* <0.01 and three (***) for *P* <0.001.

Levels of total Tau, p-Tau, Aβ 1-40 and Aβ1-42 in the CSF of AD and rpAD patients were determined by ELISA. We ensured the stability of these biomarker proteins to defined short-term storage conditions [see Additional file 2]. Our data revealed that CSF dementia marker profiles, as well as the general Aβ-40/42 ratio, did not vary significantly between AD and rpAD patients, suggesting an independent role of the immune system responses in rpAD (Figure 3A-E).

## Discussion

Rapidly progressive dementia, in contrast to the more common forms of dementia, is a subset that follows an accelerated course of cognitive, behavioral and motor decline over a period of less than 2 years. An important cause is CJD or rpAD.

We aimed to understand the role of the immune system as a modifier of disease pathogenesis in AD with various progression rates.

Performing a multiplex analysis of 17 cytokines and chemokines in serum and CSF, we analyzed inflammatory profiles of rapidly progressive dementias such as CJD and rpAD (disease duration <2 years) in comparison to ‘classical’ AD as a form of dementia with a general disease duration of 7 to 20 years. Furthermore, we included patients with MS as a candidate disease for a chronic inflammatory CNS disease with neurodegeneration. Interestingly, we observed a characteristic proinflammatory cytokine response in rpAD patients, which was not obtained in the same way as in the classical AD patients. Several proinflammatory cytokines were elevated in the serum of rpAD patients, which may reflect a genuine systemic immune response to be causative for the rapidly progressive course in this AD subform.

### Proinflammatory immunomarkers are elevated in the serum of patients who have a rapidly progressive form of Alzheimer’s disease

RpAD was recently identified as representing a rapid subtype of AD [13]. In comparison to other dementias (AD or CJD), MS and controls, we observed an elevated level of proinflammatory cytokines (IL-6, IL-13, TNF-α and G-CSF) specifically in the serum of rpAD patients. This cytokine spectrum reflects both a Th1 and Th2 immune response. However, the spectrum weighs in favor of a proinflammatory immune activation. The level of PrP^C^ did not vary significantly between AD and rpAD either in the serum or in the CSF, indicating that the increased immune response in rpAD occurs independently of PrP^C^. This observation is in line with a previous study, which demonstrated that the PrP^C^ level in CSF is not associated with the cognitive status of AD patients [21].

To our knowledge this is the first study providing an inflammatory profile for rpAD patients in comparison to AD patients. Findings of previous studies on AD patients are contradictory, leaving the debate of inflammatory marker in AD still open. While *Licastro et al*. [22] *and Sun et al.* [23] observed an increase inflammatory response in the blood of AD patients, other goups reported that immunological responses are not the major contributors to the pathogenesis of AD [24,25]. This discrepancy may reflect different detections assays with different sensitivities or different patient numbers and characteristics.

Our finding might reflect a systemic immune activation in the rpAD group that did not present in the same way in MS, AD or CJD and control patients. It may be influential in the acceleration of the disease course in AD, independent of the general phenomenon in rapidly progressive dementia pathogenesis because in CJD the immunomarkers were not elevated in the same way. This argument is supported by the finding that we do not see major differences among the CSF destruction markers Tau, p-Tau and Aβ1-40/-42 or between the Aβ ratios in AD and rpAD patients.

Our findings may be helpful in explaining pathogenetic differences between rpAD and AD.

### Elevated levels of IL-8 und MCP-1 in the cerebrospinal fluid of Creutzfeldt-Jakob disease and Alzheimer’s disease patients

Since CSF has been demonstrated to be a subanalyte for the detection of biomarkers in neurodegenerative dementia (both AD and CJD) [15] and has provided a better understanding of neurodegenerative brain diseases, we screened the CSF in patients with rapidly progressive dementias such as CJD or rpAD for inflammatory markers.

Interestingly, we found elevated levels of the chemokines IL-8 and MCP-1 in the CSF of CJD patients and MCP-1 in the CSF of AD patients. As these markers are mostly involved in mechanisms of the innate immune response, they might represent an atypical neuroinflammatory response as reflected by microglia activation and astrogliosis in the neurodegenerative process. Previous studies on inflammatory markers in the CSF of AD patients are also conflicting. Most of the groups observed no marked differences between AD and the control group [25], which is in line with our findings. An increased level of MCP in AD patients was described in blood earlier [26].

From the rapidly progressive dementia group, IL-8 and MCP-1 were only elevated in the CSF of CJD but not in rpAD patients, indicating a more disease-specific function independent from the course of the disease. Increased levels of IL-8 in the CSF of CJD patients are in line with a previously reported study using a regular ELISA [27].

Discrepancies, observed in results of divergent cytokine levels in the CSF and serum, are most likely related to the blood brain barrier, which separates the brain’s immune system from the periphery. The blood brain barrier is partly responsible for the initial concept that the brain is an immune privileged site, with restricted passage of immune cells into the brain. It prevents the passage of large molecules from the blood into the brain parenchyma [28]. It is therefore assumed that the CSF directly mirrors pathological events in the central nervous system (CNS); thus cytokine regulations in serum reflect peripheral immune responses independendly from the CNS.

In conclusion, an understanding of the neuroinflammatory process in neurodegenerative dementias becomes more and more important, especially as it might reveal new insights into the pathogenesis of rapidly progressive forms of dementia (rpAD, CJD). Our major finding shows a unique immune response profile in the serum of the group of rpAD that was distinguishable from the other disease groups, especially ‘classical’ AD. Our results may indicate a systemic activation of the immune system in this AD subtype.

## Additional files

Additional file 1:
**Statistical analysis of cytokine levels in CSF and serum.** For all patient groups, mean and SD were shown for IL-6, IL-7, IL-8, IL-12, IL-13, MCP-1, MIP-1 beta, G-CSF and TNF-α. Additional file 2:
**Stability of Aβ1-40, Aβ1-42, Tau and p-Tau 181 under defined storage conditions.** CSF samples from AD patients (n =8) were stored at -80°C, and incubated at 4°C, as well as at room temperature (RT), for 7 days. Protein expression levels were determined by ELISA and calculated as percent of control samples at time point zero, defined as 100%. No significant decrease in (A) Aβ1-40, (B) Aβ1-42, (C) Tau and (D) p-tau 181-level could be detected under these short-term storage conditions. Error bars represent means with SD. The number of stars indicates the significance level: one star (*) for *P* <0.05, two (**) for *P* <0.01 and three (***) for *P* <0.001.

## Abbreviations

Aβ: amyloid beta
AD: Alzheimer’s disease
CJD: Creutzfeldt-Jakob disease
CSF: cerebrospinal fluid
MS: multiple sclerosis
PrP: prion protein
rpAD: a rapidly progressive form of Alzheimer’s disease
