# Supplementary figures and images for: Immune responses in rapidly progressive dementia: a comparative study of neuroinflammatory markers in Creutzfeldt-Jakob disease, Alzheimer’s disease and multiple sclerosis
Source: J Neuroinflammation. 2014 Oct 15;11:170. doi: 10.1186/s12974-014-0170-y (PMC4207356; doi:10.1186/s12974-014-0170-y)

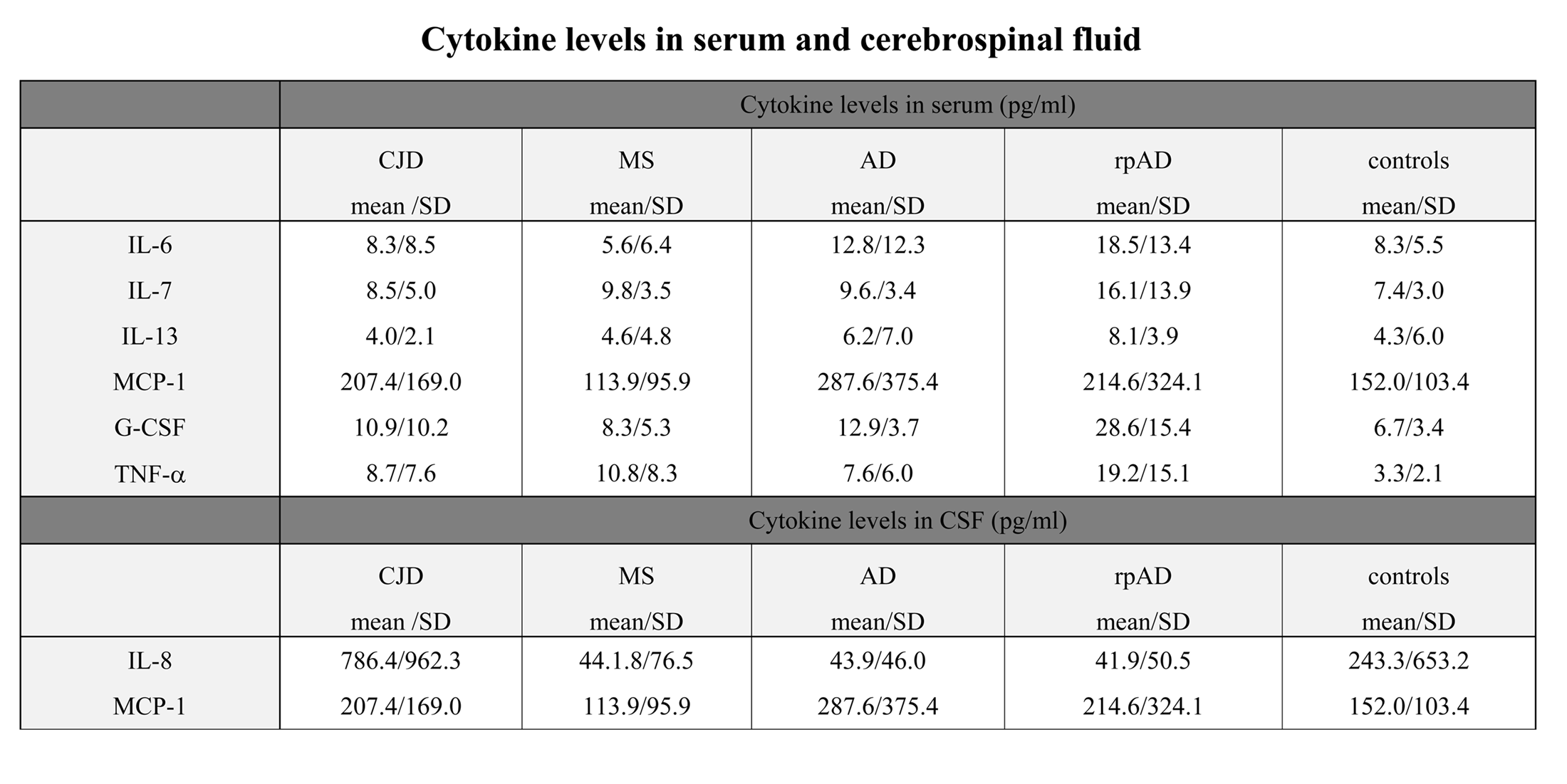

Supplement: Additional file 1: — Statistical analysis of cytokine levels in CSF and serum. For all patient groups, mean and SD were shown for IL-6, IL-7, IL-8, IL-12, IL-13, MCP-1, MIP-1 beta, G-CSF and TNF-α. [file 12974_2014_170_MOESM1_ESM.tif]

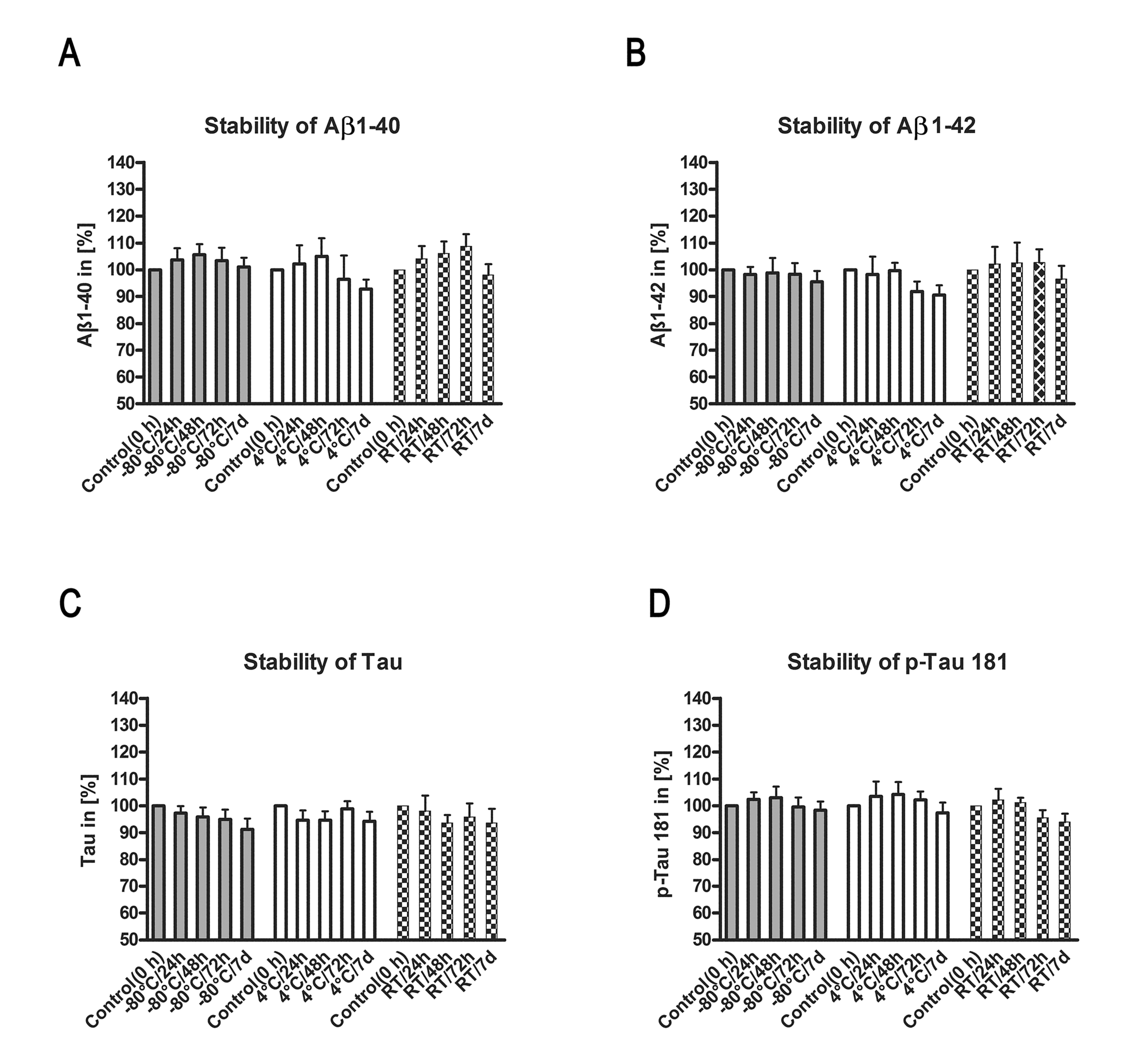

Supplement: Additional file 2: — Stability of Aβ1-40, Aβ1-42, Tau and p-Tau 181 under defined storage conditions. CSF samples from AD patients (n =8) were stored at -80°C, and incubated at 4°C, as well as at room temperature (RT), for 7 days. Protein expression levels were determined by ELISA and calculated as percent of control samples at time point zero, defined as 100%. No significant decrease in (A) Aβ1-40, (B) Aβ1-42, (C) Tau and (D) p-tau 181-level could be detected under these short-term storage conditions. Error bars represent means with SD. The number of stars indicates the significance level: one star (*) for P <0.05, two (**) for P <0.01 and three (***) for P <0.001. [file 12974_2014_170_MOESM2_ESM.tif]
